# Safety Evaluation of Oleoresin-Based Turmeric Formulation: Assessment of Genotoxicity and Acute and Subchronic Oral Toxicity

**DOI:** 10.1155/2022/5281660

**Published:** 2022-03-31

**Authors:** Somashekara Nirvanashetty, Sanjib Kumar Panda, Shavon Jackson-Michel

**Affiliations:** ^1^- Olene Life Sciences Private Limited, Chennai, 600058 Tamil Nadu, India; ^2^Dolcas Biotech, LLC 9 Lenel Road, Landing NJ 07850, USA

## Abstract

Turmeric rhizome (Curcuma longa L.) has been used without concern for safety as a culinary spice and traditional medicine under the ancient Ayurvedic medicinal system of India dating back nearly 4000 years. This preclinical safety evaluation was done to determine the safety of an oleoresin-based turmeric extract (CURCUGEN®). Guidelines from the Organization for Economic Co-operation and Development (OECD) directed the assessment of safety for the *in vitro* and *in vivo* application of CURCUGEN®. Safety of the herbal medicine was evaluated through the toxicological assessment of acute, oral, and 90-day repeated dosing, genotoxicity, and mutagenicity study. Genotoxicity tests included the *in vitro* bacterial reverse mutation test, chromosomal aberration test, and *in vivo* micronucleus test. The single dose of CURCUGEN® administered orally (gavage) to Sprague-Dawley (SD) rats resulted in a LD50 of >5000 mg/kg body weight. The subchronic assessment of CURCUGEN®, as administered to SD rats over 90 days resulted in a no observed adverse effect level (NOAEL) of 2000 mg/kg body weight/day. CURCUGEN® did not elicit any genotoxic or clastogenic effect in genotoxicity tests. The battery of safety studies carried out demonstrated that CURCUGEN® showed no evidence of general toxicity or genotoxicity.

## 1. Introduction

Turmeric is a yellow-colored culinary spice that has received much interest in the medical world due to its potential health benefits and history of safe use. It is shown to have an array of health benefits and has a traditional medicine use history, under the ancient Indian medical system (Ayurveda), that is thousands of years long [1]. The range of pharmacological activity of curcumin is varied and inclusive of antioxidant, anti-inflammatory, antiarthritic, antiangiogenic, antitumor, antiulcer, and antiaging properties. It has been shown to benefit metabolic syndrome and possesses neuroprotective activity. The health benefits of turmeric are attributed to the presence of yellow-colored polyphenolic compounds called curcuminoids. Curcuminoids are a group of related polyphenolic compounds mainly consisting of curcumin and its minor analogues, demethoxycurcumin (DMC) and bisdemethoxycurcumin (BDMC) [1, 2]. They are also shown to be anticarcinogenic, antimicrobial, and anti-inflammatory [3, 4].

Literature review of the last decade has clearly indicated that a lot of research has been done on curcumin extracts made using different formulation methods. The different extracts formulated by various methods and additives have been analyzed for their pharmacological efficacy and safe use, by way of preclinical safety experiments and safety end points used in clinical trials.

Several preclinical and clinical studies demonstrated that curcuminoids are safe even when consumed in high doses. Preclinical safety studies have determined curcumin nonclastogenic and nonmutagenic in nature and without reproductive toxicity when orally administered [5]. Oral dosing regimens of 5000 mg/kg and up to 1000 mg/kg body weight per day in single-dose acute and subchronic repeat-dose toxicity studies, respectively, have further evidenced curcumin to be without general toxicity [6–10]. The tolerance of standardized (e.g. 95% concentrated) curcumin extract in a dose escalation study on healthy subjects was found to be excellent up to 12 g, as a single oral dose [11].

CURCUGEN®, an oleoresin-based turmeric extract standardized to 50% curcuminoids, and inclusive of turmeric essential oils and turmeric polysaccharides, is being developed as an herbal medicine. In a randomized, 2-arm double-blind, placebo-controlled study on middle-aged men and women with self-reported digestive complaints, CURCUGEN® effectively reduced Gastrointestinal Symptom Rating Scale (GSRS) and anxiety scores without eliciting severe or serious adverse events [3]. The study presented here was done to evaluate the safety of CURCUGEN® through a standard sequence of preclinical tests, inclusive of acute toxicity; subchronic, oral, repeat-dose 90-day toxicity, and genotoxicity studies. The studies were performed in a GLP-certified lab and Committee for the Purpose of Control and Supervision on Experiments on Animals- (CPCSEA-) approved test facility at Bioneeds India Private Limited, located at Devarahosahally, Sompura Hobli, Nelamangala Tq., Bangalore Rural District–562111, Tumakuru, India. Each study was approved by the Institutional Animals Ethics Committee (IAEC) at Bioneeds India, and all ethical practices for animal care were followed. Organization for Economic Co-operation and Development (OECD) guidelines for each test were followed for alignment to global regulatory standards.

## 2. Materials and Methods

### 2.1. Test Sample

CURCUGEN® is a standardized curcuminoid extract with 50% curcuminoid, 1.5% essential oils, and other constituents of turmeric manufactured by Olene Life Sciences Pvt. Ltd.

### 2.2. Chemical and Bacterial Strains Used

For the genotoxicity studies, positive controls were acquired from Sigma Aldrich (2-aminoanthracene, 9-aminoacridine, sodium azide, and cyclophosphamide monohydrate), Tokyo Chemical Industry (2-nitrofluorene), and MP Biomedicals (mitomycin C). Fetal bovine serum (FBS) was procured from Gibco. RPMI-1640 media was procured from MP Biomedicals. *Salmonella* strains were procured from Moltox, USA, and S9 fractions were prepared fresh, in-house.

### 2.3. Bacterial Reverse Mutation Test (Ames Test)

CURCUGEN® was evaluated for mutagenicity in bacterial reverse mutation test as per the OECD guideline for testing of chemicals no. 471, “bacterial reverse mutation test”, adopted on 21st July [12]. *Salmonella typhimurium* strains TA98, TA100, TA102, TA1535, and TA1537 were used in this study. The study was conducted in the presence and absence of the external metabolic activation system. The S9 rat liver metabolic activation was prepared fresh, in-house. The mutagenic potential was determined using two procedures, namely, preincubation and plate incorporation methods. Dimethyl sulfoxide (DMSO) was used as a vehicle and CURCUGEN® was found soluble at 50,000 *μ*g/mL. For each concentration of test items, vehicle and positive control, triplicate plates were used in the study. Positive controls were used for both the presence and absence of a metabolic activation system. The selected positive controls were 2-nitroflurene, sodium azide, mitomycin C, and 9-aminoacridine for TA98, TA100 and TA1535, TA102 and TA1537, respectively. In the study on metabolic activation, 2-aminoanthracene was used as a positive control.

A preliminary study to assess the cytotoxicity of CURCUGEN® and to select the concentrations to be used in the main study was conducted using *Salmonella typhimurium* strain TA100. A fresh culture of bacteria was grown up to the late exponential or early stationary phase of growth. The inoculum was adjusted to a density of 18 × 10^8^ cells/mL. In the initial cytotoxicity test, *Salmonella typhimurium* TA100 tester strain was exposed to concentrations of 6.25, 12.5, 25, 50, 100, 200, 400, 800, and 1600 *μ*g/plate of test item in triplicate, both in the presence and absence of metabolic activation, and along with concurrent vehicle control (DMSO). Based on the results of the initial cytotoxicity test, which resulted in cytotoxicity with lawn intensity (1+) at 1600 *μ*g/plate, thin lawn (2+) at 800 *μ*g/plate, and slightly thin lawn (3+) at 400 *μ*g/plate, 400 *μ*g/plate was selected as the highest testing concentration. Wherein lesser concentrations would likely evidence a thick, healthy lawn (4+), as compared to vehicle control plates, the other concentrations selected for the plate incorporation method and preincubation method were 4, 13, 40, and 130 *μ*g/plate (with half-log dose interval).

The plate incorporation method was carried out with test concentrations 4, 13, 40, 130, and 40 *μ*g/plate of the test item, vehicle control, and positive control. While it is standard to test up to 5000 *μ*g/plate, 1600 *μ*g/plate was selected as the highest test concentration given the heavy precipitation and lawn evaluation interference resulting from the 3200 and 5000 *μ*g/plate concentrations. Only mild to minimum precipitation occurred between 1600 *μ*g/plate and 400 *μ*g/plate and no precipitation from 6.25 to 200 *μ*g/plate. The tester strains along with S9/phosphate buffer saline were mixed with 2 mL soft agar and poured onto minimal glucose agar plates. Five concentrations of the test items were plated with each of the following tester strains of *Salmonella typhimurium* TA98, TA100, TA102, TA1535, and TA1537 with and without metabolic activation. Plates were incubated at 37 ± 1°C for 64 h and 25 min. In the preincubation method, the tester strains along with S9/phosphate buffer saline were mixed and incubated in an incubator shaker for 25 minutes at 100 ± 5 rpm and 37 ± 1°C. In the postincubation study, a similar method as described in the plate incorporation method was followed with the plates being incubated at 37 ± 1°C for 67 h and 30 min.

The bacterial background lawn was evaluated manually for evidence of test item cytotoxicity using the code system, and revertant colonies for each strain within the test item dilution series were counted manually.

### 2.4. *In Vitro* Chromosomal Aberration Test

Chromosomal aberration test on CURCUGEN® was performed in accordance with the OECD guidelines for testing of chemicals, no. 473, “*in vitro* mammalian chromosomal aberration test” adopted on 29^th^ July [13] and in accordance with the recommendation of the Indian Council of Medical Research guidelines on biomedical research on human participants. The study was approved by the Institutional Ethics Committee (IEC) (IEC protocol no. BIO-IEC 169, approved on 05.09.2019).

Human peripheral lymphocytes were collected from the blood of healthy, young, nonsmoking male donors (28, 25, and 27 years of age) with no known recent exposure to genotoxic chemicals or radiation and used for the study.

DMSO was used as a vehicle and CURCUGEN® at a 1000 *μ*g/mL was found to be a suspension with moderate precipitation during incubation. Hence, 1000 *μ*g/mL was selected as the highest concentration for testing in the initial cytotoxicity test. The other concentrations selected were 62.5, 125, 250, and 500 *μ*g/mL of the test items. As there was cytotoxicity at all tested concentrations, a follow-up cytotoxicity test was performed with the lower doses 1.95, 3.9, 7.8, 15.6, and 31.25 *μ*g/mL to assess the cytotoxicity of the test item.

The initial and follow-up cytotoxicity tests are briefly described. Whole blood of volume 0.5 mL was added to each tube containing culture media of volume 4.5 mL with 2% phytohemagglutinin (PHA) and incubated for 44 to 48 h at 37 ± 1°C and 5 ± 1% CO2. The cells in the tubes were centrifuged at 1500 rpm for 10 min. The cell pellet was resuspended with 2 mL fresh culture media. The two tests were done in three sets with and without metabolic activation. Set 1 of both tests was with metabolic activation. The cell suspension was mixed with 50 *μ*L each of the respective test concentrations/vehicle and 0.5 mL of S9 mix; the volume was made up to 5 mL with culture media. Sets 2 and 3 were without metabolic activation, wherein the cell suspension was mixed with 50 *μ*L each of the respective test concentrations/vehicle and the volume was made up to 5 mL with culture media. CURCUGEN® and control concentrations were maintained in duplicate. Cells from sets 1 and 2 were incubated for 3 to 6 h and set 3 for 20 to 24 h at 37 ± 1°C and 5 ± 1% CO2. The treatments for set 1 and 2 tubes were terminated post 3 to 6 h of incubation, by centrifugation at 1500 rpm for 10 min. The precipitate was mixed with 5 mL of fresh culture medium and incubated further to complete 20 to 24 h, starting from the onset of treatment. The treatments for set 3 tubes were terminated after 20 to 24 h of incubation, by centrifugation at 1500 rpm for 10 min. Before 1 to 3 h of harvesting, colchicine of concentration 0.3 *μ*g/mL was added to the tubes of sets 1, 2, and 3. Postincubation of 1 to 3 h with colchicine, the cell suspension was collected into prelabeled tubes and centrifuged for 10 min at 1500 rpm. Pellets were mixed with 3 to 4 mL of freshly prepared warm 0.56% potassium chloride. The cell suspension was incubated for 10 min at ambient temperature and then centrifuged at 1800 rpm for 10 min. The supernate was removed leaving the cell pellet to be mixed with 3 mL of a freshly prepared cold acetic acid : methanol fixative (1 : 3). The cell suspension was incubated for 10 additional minutes at room temperature and later centrifuged at 2200 rpm for 10 min. The procedure was repeated twice by adding 3 mL of cold acetic acid : methanol fixative (1 : 3). Prechilled slides were taken, and the cell suspension was mixed using a pipette and a few drops of the suspension were aspirated and dropped onto the slide. The slides were air dried. A minimum of 3 slides were prepared for each treatment replicate. Slides were stained using 5% Giemsa stain for 15 min.

Percent mitotic index (MI %) was determined by the following formula:

(1) $$\begin{array}{c} \text{Percent mitotic index}=\text{Number of Mitotic cells}\times 100\text{ Total number of cells scored}. \end{array}$$

The percent reduction in mitotic index was obtained by using the formula:

(2) $$\begin{array}{c} \text{Percent reduction in mitotic index}=\left[\frac{\left(\text{Percentage MI of VC}-\text{Percentage MI of treated}\right)}{\text{Percentage MI of VC}}\right]\times 100, \end{array}$$

where VC is the vehicle control and MI is the mitotic index.

Concurrent measures of the mitotic index for all treated and vehicle control cultures were determined. Cytotoxicity results, determined by calculating the percent reduction in mitotic index (%), informed the low, mid, and high chromosomal aberration test concentrations of 1.95, 3.9, and 7.8 *μ*g/mL, respectively. A minimum of 300 well-spread metaphases for each of the concentrations, 1.95, 3.9, and 7.8 *μ*g/mL were analyzed. SPSS software version 22 was used to assess for differences between the vehicle control, cyclophosphamide monohydrate positive control, and CURCUGEN® groups using an analysis of variance with post hoc Dunnett's test at a 95% level of confidence (*P* < 0.05).

### 2.5. *In Vivo* Micronucleus Test

CURCUGEN® was evaluated for genotoxic potential through in vivo micronucleus test as per OECD guideline no. 474, “mammalian erythrocyte micronucleus test”, adopted on 29^th^ July [14].

The studies were approved by the Institutional Animals Ethics Committee (IAEC) of the test facility (protocol no. BIO-IAEC 3752, approved on 08/08/2019). The test facility is approved by the Committee for the Purpose of Control and Supervision on Experiments on Animals (CPCSEA), India. The studies were performed following all ethical practices as laid down in the guidelines for animal care.

The prestudy (dose range finding study) consisted of four groups, vehicle control, 500 mg/kg body weight, 1000 mg/kg body weight, and 2000 mg/kg body weight of CURCUGEN®, respectively. The test items and vehicle control were dosed for two consecutive days by oral route using oral gavage cannula. The prestudy grouping consisted of 3 male and 3 female mice—one of the recommended species of rodents acceptable among regulatory agencies for the in vivo micronucleus testing. There was no observable toxicity in the prestudy for the tested doses; hence, 2000 mg/kg body weight was selected as the limit dose for the main study. The main study consisted of 3 groups of mice, and each group consisted of 5 males and 5 females. In the main study, control group animals were administered with vehicle, G2 animals were administered with positive control (cyclophosphamide monohydrate) at the dose of 100 mg/kg body weight, and G3 group animals were administered with 2000 mg/kg body weight of CURCUGEN®—each for two consecutive days by oral route using an oral gavage cannula. Post 18 to 24 h of the last dosing, all mice were sacrificed by cervical dislocation and all animals were subjected to gross pathological examination.

The femur was isolated from each animal for bone marrow collection. Bone marrow cells were obtained by cutting open the epiphyses of femur bone, immediately following sacrifice. The marrow was flushed out into a centrifuge tube using the fetal bovine serum (FBS). The femur bone marrow cells were centrifuged at about 2700 rpm for 10 min. Prior to smear preparation, the supernatant was discarded, and the cell pellet was then resuspended in approximately 50 *μ*L of FBS.

A minimum of three slides per animal were prepared in both the prestudy and main study. Prestained smears were fixed by immersing the slides in methanol for approximately 5 min. The air-dried slides were stained with May-Gruenwald and Giemsa stain for evaluation. In the dose range finding study for each animal, a minimum of 500 erythrocytes (which include mature and immature erythrocytes) were scored to determine the polychromatic erythrocytes (PCE) and normochromatic erythrocyte (NCE) ratio, to determine PCE : total RBC ratio. This result was used to determine the cytotoxicity of the test item.

In the main study, for each animal, a minimum of 500 erythrocytes (which included mature and immature erythrocytes) were scored from the first slide of the animal to determine PCE : total RBC ratio along with the incidence of micronucleus. The subsequent slides were scored only for the number of PCEs and incidence of micronucleated PCEs.

For each animal, a minimum of 4000 polychromatic erythrocytes (PCEs) were scored for the incidence of micronucleated polychromatic erythrocytes (MNPCEs). The data of positive control and treatment groups were compared with that of the vehicle control for the incidence of MNPCEs and the proportion of PCEs among total RBCs (red blood corpuscles) by SPSS (Statistical package for Social sciences) at a 95% level (*P* ≤ 0.05) of significance. All analysis and comparisons were evaluated at the 95% level of confidence (*P* < 0.05).

### 2.6. Acute Oral Toxicity Test

CURCUGEN® was evaluated for acute oral toxicity in Sprague-Dawley (SD) rats as per OECD guidelines no. 425, by the conduct of “acute oral toxicity-up-and-down-procedure (UDP)” adopted on 3 October [15].

Earlier acute oral toxicity studies in Wistar rats and Swiss Albino mice wherein the curcumin products dosed at 5000 mg/kg body weight did not show any signs of toxicity [6, 7], and hence, 5000 mg/kg body weight was selected as testing dose for acute oral toxicity study with CURCUGEN®. The test was conducted with three animals and the three animals were sequentially dosed at 5000 mg/kg of body weight. At the previously published, safety-presumed dose of 5000 mg/kg body weight of this botanical extract, tolerability, and survivability were assessed [6–8]. The results were evaluated as per acute oral toxicity (OECD guideline 425) Statistical Programme (AOT 425 Statpgm) version: 1.0, 2001. By default of the guidelines followed to evaluate at an equivalent dose, the LD_50_ was determined to be >5000 mg/kg body weight.

The animals were observed for clinical signs of toxicity at 20 to 30 min,1 h (±10 min), 2 h (±10 min), 3 h (±10 min), and 4 h (±10 min) postdosing on day 1 and thereafter once daily for clinical signs of toxicity and twice daily for mortality during the 14-day observation period. Gross pathology was done at the end of the study period.

### 2.7. Repeated Dose 90-Day Oral Toxicity Test

The OECD guideline no. 408, “repeated dose 90-day oral toxicity study in rodents” [16] provided the regulatory outline for evaluating the toxicological profile for CURCUGEN®'s use over an extended period.

A total of 60 SD rats (30/sex), aged at 7 weeks old at the time of treatment, were grouped by body weight (Table 1) and randomly allocated to 8 groups, stratified by gender to be assessed after 90 days of treatment (main group) or to be followed for an additional 28 days, post-treatment (recovery group). Ten rats/gender were allocated to each main group, and five rats/gender were allocated to each recovery group. The body weight variation of the animals selected for the experiment did not exceed ±20% of the average body weight of each gender (male -5.53% to +6.52% and female -4.36% to +4.94%).

Altromin 1324, an alfalfa and soy-free cereal-based formula was selected as an appropriate maintenance diet for the rats throughout the acclimatization and experimental periods. The rats were given *ad libitum* access to both Altromin and reverse osmosis water throughout the experimental period.

Carboxy methyl cellulose (CMC, 0.5% *w*/*v*) was used as vehicle and test item, CURCUGEN® was prepared as suspension in 0.5% w/v CMC. A control group was included, which was administered with vehicle alone. The limit dose of 2000 mg/kg/day was selected for CURCUGEN® per OECD 408 guidelines describing the use of a test at one dose level of at least 1000 mg/kg of body weight/day for nontoxic compounds not expected to produce any observed adverse effects given previous data [7, 9, 10]. The limit test precludes the need for full study of three dose levels. Since the toxicity of curcumin is well characterized and detailed toxicity data is publicly available, CURCUGEN® being a naturally extracted curcumin/turmeric ingredient, no dose-range finding study was conducted and the study design was limited to a single dose of 2000 mg/kg which is the maximum dose allowed in repeated dose toxicity studies [6, 7]. Furthermore, given that the test item is not considered to be a new compound and toxicity and toxicokinetic data is readily available in the public domain, TK/PK evaluation was not considered a part of this study.

The test formulations/vehicle was administered through oral gavage using an oral intubation cannula attached to a disposable syringe. The animals were dosed with 10 mL/kg of vehicle or CURCUGEN® once a day for a period of 90 consecutive days.

Following the 90-day treatment period, the recovery group animals were not given any treatment and maintained for an additional 28 days to be observed for reversibility or persistence of toxic effects.

The rats were surveyed for clinical signs of toxicity once daily, and morbidity/mortality twice daily during the treatment and recovery periods. Detailed clinical examinations were performed at study commencement before initial dosing and at weekly intervals until the end of the study.

Body weight for each animal was recorded on day 1, before study treatment initiation, and weekly thereafter. Individual animal feed consumption was recorded weekly, as the sum of the spillage plus residual feed, deducted from the total amount of feed given, divided by the total by the number of rats per cage. The fasting body weights of all animals were recorded at terminal sacrifice.

Ophthalmoscopic and neurological/functional assessments were carried out during week 13 for main group animals and during week 17 for recovery group animals. Blood samples were collected from the rats on experimental days 91 and 119 for the main and recovery groups, respectively. Blood samples were collected from the animals into tubes containing K2-EDTA for hematology analysis, sodium heparin tubes for clinical chemistry, and sodium citrate tubes for prothrombin time (PT) and activated partial thromboplastin time (APTT). The plasma was separated by centrifuging the blood samples at 5000 rpm for 10 min for determining the clinical chemistry and 1500 rpm for 15 min for determining the PT and APTT parameters.

The Advia Hematology System 2120 (Siemens Limited) was used to estimate: hemoglobin concentration (HGB), hematocrit (HCT), erythrocyte count (RBC), total leukocyte count (WBC), mean corpuscular volume (MCV), mean corpuscular hemoglobin (MCH), mean corpuscular hemoglobin concentration (MCHC), platelet count (PLT), mean platelet volume (MPV), reticulocyte count (Retic), absolute reticulocyte count, differential leucocytes count (DLC), and absolute differential leucocytes count (absolute DLC). The coagulation analyzer by Tulip Diagnostics (p) Ltd., India, was used to estimate the clotting parameters, (PT) and (APTT).

The following clinical chemistry parameters were analyzed using the “Rx Daytona^+^ Clinical Chemistry Analyzer” (Randox Laboratories): alanine aminotransferase (ALT), aspartate aminotransferase (AST), alkaline phosphatase (ALP), cholinesterase, total protein, albumin, total bilirubin, glucose, total cholesterol, creatinine, urea, triglycerides, phosphorous, calcium, high-density lipoprotein (HDL), low-density lipoprotein (LDL), blood urea nitrogen (BUN, calculated), globulin (calculated), albumin/globulin ratio (calculated). sodium (mmol/L), potassium (mmol/L), and chloride (mmol/L) were estimated using a Na/K/Cl analyzer (Diamond Diagnostics/Medica Corporation).

Overnight urine samples were collected from all rats of main and recovery group animals on experimental day 91 and 119, respectively, to perform urinalysis. Physical parameters including urine volume (mL), appearance, and color were evaluated by visual inspection and recorded. The urine analyzer Dirui H-500 (Dirui Industries) assessed parameters of blood, bilirubin, urobilinogen, ketones, protein, glucose, leucocytes, and microalbumin in the urine. pH, nitrite, and specific gravity were additionally analyzed. The postanalysis urine was subjected to centrifugation at 1500 rpm for three minutes, readying it for microscopic examination of residual elements.

An assessment of thyroid hormones, T3 (triiodothyronine), T4 (thyroxine), and thyroid-stimulating hormone (TSH) was also done. The blood samples for the estimation of T3, T4, and TSH were collected at termination and centrifuged at 5000 rpm for 10 min, and the separated serum was prepared for freezer storage at -80°C.

All animals in the main group were necropsied on day 91, and a thorough appraisal of possible gross pathological changes was performed. On day 119, the animals in the recovery group were sacrificed and, respectively, necropsied. External surfaces, external orifices, abdominal, thoracic, and cranial cavities, organs, and tissues were examined as part of the postmortem evaluation.

Upon dissection of both control and CURCUGEN® animals, their livers, lungs, kidneys, spleens, adrenals, thymus', hearts, brains, prostate glands, testes, epididymis, ovaries, uteruses, thyroids, and parathyroid, pituitary glands were extracted, pared of excess connective tissue, weighed, and independently fixed in 10% neutral buffered formalin solution for histopathological examination. A modified Davidson's fixative was used to support the microscopic examination of the animal's eyes and testes.

Testes were sectioned at 3-4-micrometer thickness and in addition to hematoxylin and eosin stain, periodic acid-Schiff, and hematoxylin stain was used for spermatogenesis evaluation.

An in-depth qualitative examination of the testes was conducted to identify treatment-related effects in the spermatogenic cycle such as missing germ cell layers or types, retained spermatids, multinucleate or apoptotic germ cells, and sloughing of spermatogenic cells into the lumen or any other pathological cell or stage-specific testicular findings.

Vaginal smear was examined from the females on the day of sacrifice to determine estrous cycle stages.

SPSS statistical analysis software, version 22, was used to examine differences in body weight, percent change in body weight—as compared to first-day data, feed consumption, organ weight and ratio, hematological and clinical chemistry estimations, urinalysis parameters (urine volume, pH, specific gravity, and urobilinogen), and FOB parameters (rearing, urination, defecation, excessive grooming, body temperature, grip strength, motor activity, and hind limb foot splay) control and CURCUGEN® groups after 90 days of dosing and after an additional 28 days of recovery. Using Student's ‘*t*' test all comparisons were evaluated at the 95% level of confidence for significance.

## 3. Results

### 3.1. Bacterial Reverse Mutation Test (Ames Test)

CURCUGEN® was evaluated for genotoxicity using bacterial reverse mutation test at the concentrations of 4, 13, 40, 130, and 400 *μ*g/plate incorporation method (Table 2) and preincubation method (Table 3) using *Salmonella typhimurium* TA98, TA100, TA102, TA1535, and TA1537.

In both the plate incorporation method and preincubation method, the test item resulted in no appreciable increase in the number of revertant colonies over the vehicle control, while the positive controls (2-nitrofluorene, 2-aminoanthracene, 9-aminoacridine, sodium azide, and mitomycin C) tested simultaneously resulted in 2.3- to16.9-fold increase in the number of revertant colonies/plate under identical conditions (Tables 2 and 3). Similar results were observed for all five tester strains.

Bacterial reverse mutation investigations established CURCUGEN® as nonmutagenic under the conditions tested.

### 3.2. *In Vitro* Chromosomal Aberration

The reduction in mitotic index (MI) observed for CURCUGEN® at the concentrations of 7.8 *μ*g/mL was 46.50% with metabolic activation and 42.41% without metabolic activation for short-term treatment. Similarly, the reduction in MI observed for CURCUGEN® at the concentrations of 7.8 *μ*g/mL was 45.23% without a metabolic activation system for long-term treatment.

CURCUGEN® did not induce statistically significant (*P* < 0.5) increases in the number of aberrated cells when compared with vehicle control.

The observed mean percent aberrated cells at the concentrations of 1.95, 3.9, and 7.8 *μ*g/mL in the presence of metabolic activation (short-term treatment 3 to 6 h) were 1.33, 1.67, and 1.34, respectively. Similarly, the observed mean percent of aberrated cells at the concentrations of 1.95, 3.9, and 7.8 *μ*g/mL in the absence of metabolic activation (short-term treatment 3 to 6 h) were 1.33, 1.34, and 1.67, respectively. The observed mean percent aberrated cells at the concentrations of 1.95, 3.9, and 7.8 *μ*g/mL in the absence of metabolic activation for long-term treatment (20 to 24 h) were 1.67, 1.34, and 1.67, respectively.

Positive control, 10 *μ*g/mL of cyclophosphamide monohydrate, in the presence of metabolic activation (3 to 6 h), induced 11.00% of aberrated cells which was statistically significant compared to the vehicle control where it was 1.33%. The reduction in MI was 7.95% when compared with the vehicle control for short-term treatment.

Positive control, 0.05 *μ*g/mL of mitomycin C, in the absence of metabolic activation (3 to 6 h) induced 10.34% of aberrated cells, which was statistically significant to the vehicle control (1.00%). The reduction in MI observed was 8.83% when compared with the vehicle control for short-term treatment. Positive control, 0.05 *μ*g/mL of mitomycin C, in the absence of metabolic activation (20 to 24 h) induced 10.34% of aberrated cells, which was statistically significant to the vehicle control (1.33%). The reduction in MI observed was 5.97% when compared with the vehicle control for long-term treatment (data not shown).

The test results confirmed that CURCUGEN® is nonclastogenic under the test conditions.

### 3.3. In Vivo Micronucleus Test

CURCUGEN® was assessed for mammalian erythrocyte micronucleus test at 2000 mg/kg body weight in male and female Swiss Albino mice. The average percentage of micronucleated polychromatic erythrocytes (PCEs) was 0.06 in both males and females dosed. There was no significant difference in the percentage of micronucleated PCEs scored for a minimum of 4000 PCE in comparison with the vehicle dosed animals. In the positive control animals, the average percentage of micronucleated PCEs was 0.75 and 0.71 in males and females, respectively. In the positive control group, the percentage of micronucleated PCEs score was increased, which was statistically significant when compared with the vehicle control group (Table 4). This demonstrated the sensitivity of the test system towards positive control and confirmed that the test conditions were adequate.

Based on the results obtained under the conditions employed during this experiment, it is concluded that CURCUGEN® is nongenotoxic at the limit dose of 2000 mg/kg body weight.

### 3.4. Acute Oral Toxicity Test

Single oral (gavage) administration of CURCUGEN® at 5000 mg/kg body weight did not cause mortality or clinical signs of toxicity in female SD rats immediately after dosing and during the 14-day observation period. There were no test item-related gross pathological changes observed at the end of the 14-day observation period. The results indicated that the LD50 of CURCUGEN® is greater than 5000 mg/kg body weight and was found to be safe.

### 3.5. Repeated Dose 90-Day Oral Toxicity Test

CURCUGEN® was well tolerated at the dose level of 2000 mg/kg/during this repeated-dose 90-day subchronic toxicity study. No clinical signs of toxicity and mortality were observed during the study. However, dark-yellow colored urine was observed from day 8 to day 90 of the treatment period, which was reversed during the recovery period. Such a change in color of the urine is expected for naturally colored food products or supplements including curcumin products, and this change in urine color was never reported as adverse event as this is considered normal [17, 18]. There was no treatment-related variation in the mean body weight or percent body weight change with respect to day 1 in the limit dose of 2000 mg/kg/day. No treatment-related changes in feed consumption were noted in both the main group and recovery group animals.

No ophthalmological abnormalities were noted in the main and recovery group animals.

No adverse treatment-related changes were observed in the neurological/functional examination battery carried out during week 13 for the main groups (control and CURCUGEN®) and during week 17 for recovery groups (control and CURCUGEN®). However, the incidental findings of a significant decrease in urination in males, rearing in females; an increase in actimeter counts in females of the CURCUGEN® group were not considered test item related.

No adverse treatment-related changes in hematology parameters were noted. However, the observed decrease in PT in males, absolute and relative neutrophils in females, and relative basophils in females and an increase in relative lymphocytes in females of CURCUGEN® group were considered incidental and not related to the test items as no such variations were noted in other sex (Table 5). The variations noted in clinical chemistry parameters were not considered toxicologically significant (Table 6).

The observed changes in urea in males, BUN in males, sodium in males, low-density lipoprotein in females of the main and reversal group, total protein in females, albumin in females, calcium in females, and potassium in females of CURCUGEN® with no associated microscopic changes were incidental and not related to test item administration. Decrease in triglycerides in males of CURCUGEN® group was not of toxicological significance (Table 6). No toxicologically significant treatment-related changes in urinary parameters were noted.

Vaginal smear examination revealed normal stages of estrous cyclicity both in the main and recovery females of both limit dose and control group. No treatment-related changes in thyroid hormones (T3, T4, and TSH) were noted.

There were no adverse changes noted in fasting body weight, organ weights, and its ratio for males (Table 7) or females (Table 8). The changes in the absolute/relative weight of liver and epididymis are not related to the test items as there were no associated macroscopic or microscopic changes in the related organs.

No test-item-related gross pathological or histopathological findings were observed in this study.

Based on the observed results in (SD) rats ingesting CURCUGEN® at 2000 mg/kg bw/day by gavage (orally) over 90 consecutive days, no observed adverse effect level (NOAEL) of 2000 mg/kg bw/day was assessed to CURCUGEN®.

## 4. Discussion

Products of natural origin are most preferred in recent days due to their higher safety profile. Natural products are also backed with a history of safe use for several thousand years, in some cases. Turmeric is one such product with a high safety profile and history of use for nearly 4000 years as a culinary spice and traditional medicine under the ancient Ayurvedic medicinal system of India. The health benefits of turmeric are attributed to the presence of yellow-colored polyphenolic compounds called curcuminoids. Curcuminoids and curcuminoid-based products have been studied for their safety and therapeutic effect in preclinical and clinical studies alike. In preclinical safety studies, curcuminoids were found to be nonclastogenic and nonmutagenic and did not show any reproductive toxicity upon oral administration [5]. The tolerance of standardized turmeric extract in a dose escalation study on healthy subjects was found to be excellent up to 12g as a single oral dose [11]. Poor bioavailability of curcuminoids led to the development of products with enhanced bioavailability [19]. However, many of the curcuminoid-based products with enhanced bioavailability are formulated using excipients of synthetic or nonturmeric origin to improve the pharmacokinetics of curcuminoids. As per the published literature, the content of the excipients in those curcumin products are as high as 93% in some curcumin formulations and hence the safety of those excipients must be considered while evaluating the product for long-term usage [19]; [20]. It is important to determine the criticality of risk associated with the excipients versus their functionality in the formulation.

CURCUGEN® is a patent-pending, turmeric oleoresin based, 98.5% pure turmeric sourced herbal medicine, standardized to 50% curcuminoids, 1.5% retained turmeric essential oil, turmeric polysaccharides, and resins. The only excipient present in the ingredient is nonnanocategory silicon dioxide, a glidant commonly present in various food products. Unlike 95% standard curcuminoid and its formulated products, CURCUGEN® preserves the natural curcuminoids profile with fidelity to the turmeric rhizome, which makes it more natural. This preclinical safety evaluation sought to establish the safety of CURCUGEN® using a standard series of *in vitro* and *in vivo* safety studies in accordance with OECD guidelines. In the present study, CURCUGEN® did not elicit any genotoxic or clastogenic effect in genotoxicity tests. The bacterial reverse mutation test proved CURCUGEN® showed no signs of mutagenicity. As compared to vehicle control, using both plate incorporation and plate incubation methods, CURCUGEN® did not provoke a significant increase in the quantity of colonies able to reverse-mutate.

The *in vitro* chromosomal aberration test with CURCUGEN® showed it to be nonclastogenic with and without the use of metabolic activation. CURCUGEN® was nongenotoxic at the limit dose of 2000 mg/kg body weight in the *in vivo* micronucleus test.

The single dose of gavaged CURGUGEN® to SD rats showed a LD50 of >5000 mg/kg body weight per day and is in concordance with previously published LD50 values for curcuminoid products assessed in Wistar rats and Swiss Albino mice [6]; [7], [8]. The subchronic, daily dosing of CURCUGEN® in SD rats for 90 days determined a NOAEL of 2000 mg/kg body weight (bw) per day. The NOAEL of CURCUGEN® was higher than that of several curcumin formulations with enhanced bioavailability. A hydrogenated curcumin formulation in a subchronic study on SD rats was reported to have a NOAEL of 800 mg/g bw/day [9]. Another 90-day, subchronic toxicity study in Wistar rats evaluating a curcuminoid-essential oil complex, reported a NOAEL of 1000 mg/kg bw/day [7]. A similar, 90-day repeat dose study with synthetic curcumin gave a NOAEL of 1000 mg/kg bw/day [10]. Overall, the battery safety experiments carried out in this study demonstrated that CURCUGEN® is unlikely to cause toxic effects. CURGUGEN®'s close fidelity to its native turmeric raw material may have contributed to the absence of toxicity, as demonstrated by its oral administration in rats in these studies, and its appropriation to humans (i.e., NOAEL) when a minimum safety factor of 100 is applied.

## 5. Conclusion

In conclusion, CURCUGEN® is nonmutagenic and nongenotoxic and is not expected to cause adverse effects as determined from the strictly followed OECD-guided mutagenicity and genotoxicity studies. The subchronic, oral administration of CURCUGEN® for 90 days at the prescribed limit dose level of 2000 mg/kg bw/per day did not cause adverse effects in rats of either gender, resulting in an aligned NOAEL of 2000 mg/kg bw/day.

## Figures and Tables

**Table 1:** Individual weights of rats at initiation of treatment.

Group, sex, & dose (mg/kg/day)		Body weight (g) on days																	
		1	8	15	22	29	36	43	50	57	64	71	78	85	90	97	104	111	118
G1, M, & 0	Mean	160.97	198.43	238.03	265.49	294.38	323.82	346.29	363.53	377.03	392.13	400.74	402.82	408.31	414.71	—	—	—	—
	±SD	5.06	10.08	16.89	18.84	24.00	27.22	31.22	33.71	37.87	37.97	39.01	36.32	36.36	36.43	—	—	—	—
	*n*	10	10	10	10	10	10	10	10	10	10	10	10	10	10	—	—	—	—
G2, M, & 2000	Mean	161.16	188.46^∗^	222.43^∗^	251.67	278.54	309.71	333.93	344.80	359.17	370.29	377.57	373.50	382.73	389.41	—	—	—	—
	±SD	5.44	5.70	10.01	13.45	18.48	23.90	28.79	30.69	34.55	38.60	38.16	38.65	38.91	36.95	—	—	—	—
	*n*	10	10	10	10	10	10	10	10	10	10	10	10	10	10	—	—	—	—
G1, F, & 0	Mean	142.55	162.37	185.20	198.17	214.71	229.92	240.86	246.79	254.79	258.93	263.44	265.51	272.24	274.44	—	—	—	—
	±SD	4.43	6.51	9.34	13.36	12.25	12.76	12.96	14.36	15.35	16.65	16.98	17.80	16.84	17.25	—	—	—	—
	*n*	10	10	10	10	10	10	10	10	10	10	10	10	10	10	—	—	—	—
G2, F, & 2000	Mean	142.39	164.28	186.92	198.27	215.01	231.50	240.49	245.67	252.55	255.80	259.65	259.29	264.28	268.75	—	—	—	—
	±SD	3.97	7.82	6.57	7.03	7.79	6.39	8.28	10.18	14.42	15.16	15.30	15.00	13.47	13.67	—	—	—	—
	*n*	10	10	10	10	10	10	10	10	10	10	10	10	10	10	—	—	—	—
G1R, M, & 0	Mean	158.77	189.69	221.28	243.99	271.29	303.51	326.75	337.61	351.52	358.81	366.71	371.73	379.29	383.75	386.30	393.07	396.50	398.32
	±SD	5.15	11.62	16.85	19.99	24.67	24.84	30.45	36.61	42.12	45.03	44.28	40.48	37.48	33.33	31.91	30.05	30.05	30.34
	*n*	5	5	5	5	5	5	5	5	5	5	5	5	5	5	5	5	5	5
G2R, M, & 2000	Mean	159.41	191.67	230.50	253.09	280.37	309.55	334.96	342.38	356.13	368.77	373.55	375.69	387.31	393.97	396.61	406.00	412.46	415.08
	±SD	4.20	11.43	15.40	12.28	11.62	14.57	18.28	19.66	22.37	25.07	26.55	30.11	32.83	34.29	34.92	34.32	35.00	34.72
	*n*	5	5	5	5	5	5	5	5	5	5	5	5	5	5	5	5	5	5
G1R, F, & 0	Mean	140.81	159.57	183.22	194.32	213.78	227.09	234.48	239.54	243.74	245.84	248.98	250.51	256.38	260.10	261.43	265.51	266.62	267.36
	±SD	4.80	6.54	4.42	5.01	7.49	7.53	8.43	7.97	7.49	7.48	9.28	10.57	6.44	6.42	6.29	7.35	7.19	9.06
	*n*	5	5	5	5	5	5	5	5	5	5	5	5	5	5	5	5	5	5
G2R, F, & 2000	Mean	141.66	161.39	183.85	196.02	215.39	227.73	238.02	242.61	246.03	250.99	252.76	248.68	252.41	256.59	257.44	259.70	261.20	262.82
	±SD	4.91	8.18	9.45	12.24	12.60	14.92	17.40	19.82	20.44	21.48	21.82	24.08	24.07	24.71	25.42	25.85	26.22	25.80
	*n*	5	5	5	5	5	5	5	5	5	5	5	5	5	5	5	5	5	5

**Table 2:** Summary of colony counts of revertants-trial-I.

Plate incorporation method											
Treatment	Concentration (*μ*g/plate)		No. of revertants (mean of 3 plates)								
			With S9					Without S9				
			*Salmonella typhimurium*					*Salmonella typhimurium*				
			TA 98	TA 100	TA 1535	TA 1537	TA 102	TA 98	TA 100	TA 1535	TA 1537	TA 102
Vehicle control	Vehicle control (dimethyl sulphoxide)	Mean	31.0	111.7	19.3	8.3	268.7	25.3	97.7	17.3	6.7	262.3
		±SD	2.0	2.5	0.6	1.2	3.1	0.6	1.5	0.6	1.2	3.1
	Lawn intensity		4+	4+	4+	4+	4+	4+	4+	4+	4+	4+
OLNP-18	4	Mean	29.7	111.0	19.0	10.0	264.3	26.7	98.0	16.0	8.0	258.3
		±SD	1.2	1.7	1.0	1.0	5.1	1.5	3.0	1.0	1.0	2.1
	Fold increase		1.0	1.0	1.0	1.2	1.0	1.1	1.0	0.9	1.2	1.0
	Lawn intensity		4+	4+	4+	4+	4+	4+	4+	4+	4+	4+
	13	Mean	28.7	115.3	19.0	9.7	271.0	27.3	99.7	15.3	7.3	270.7
		±SD	1.5	1.5	1.0	1.5	2.6	1.5	4.7	1.5	0.6	2.5
	Fold increase		0.9	1.0	1.0	1.2	1.0	1.1	1.0	0.9	1.1	1.0
	Lawn intensity		4+	4+	4+	4+	4+	4+	4+	4+	4+	4+
	40	Mean	29.7	108.0	19.0	8.7	270.7	27.0	100.3	16.3	8.0	257.3
		±SD	1.5	3.0	1.0	1.2	3.5	2.6	1.5	1.5	1.0	2.5
	Fold increase		1.0	1.0	1.0	1.0	1.0	1.1	1.0	0.9	1.2	1.0
	Lawn intensity		4+	4+	4+	4+	4+	4+	4+	4+	4+	4+
	130	Mean	29.0	111.7	19.3	8.7	267.7	23.3	96.3	15.3	6.7	263.3
		±SD	1.0	2.5	0.6	2.1	2.5	1.2	1.5	0.6	0.6	3.2
	Fold increase		0.9	1.0	1.0	1.0	1.0	0.9	1.0	0.9	1.0	1.0
	Lawn intensity		4+	4+	4+	4+	4+	4+	4+	4+	4+	4+
	400	Mean	20.3	80.0	14.7	4.3	248.7	18.3	79.7	9.3	3.7	242.3
		±SD	1.5	1.0	0.6	0.6	1.5	1.5	1.5	0.6	0.6	3.2
	Fold increase		0.7	0.7	0.8	0.5	0.9	0.7	0.8	0.5	0.6	0.9
	Lawn intensity		3+	3+	3+	3+	3+	3+	3+	3+	3+	3+
Positive control	100 *μ*L of respective positive control	Mean	390.0	396.3	148.3	121.0	615.3	367.7	387.7	143.0	112.7	603.7
		±SD	9.2	7.4	3.1	2.0	7.5	8.1	3.1	4.0	4.7	7.4
	Fold increase		12.6	3.5	7.7	14.5	2.3	14.5	4.0	8.3	16.9	2.3
	Lawn intensity		4+	4+	4+	4+	4+	4+	4+	4+	4+	4+

3+: slightly thin lawn: distinguished by a noticeable thinning (slightly reduced) of the background lawn compared to vehicle control plates. 4+: thick lawn: distinguished by a healthy (normal) background lawn comparable to vehicle control plates. Values of revertants are in mean ± SD. Positive controls: for with S9: for *Salmonella typhimurium*, TA98, TA100, TA102, TA1535, and TA1537: 4 *μ*g/plate of 2-Aminoanthracene. For S9: for TA98: 2 *μ*g/plate of 2-nitrofluorene. For TA100 and TA1535: 1 *μ*g/plate of sodium azide. For TA102:0.5 *μ*g/plate of mitomycin C. For TA1537: 50 *μ*g/plate of 9-aminoacridine.

**Table 3:** Summary of colony counts of revertants-trial-II.

Preincubation method											
Treatment	Concentration (*μ*g/plate)		No. of revertants (mean of 3 plates)								
			With S9					Without S9				
			*Salmonella typhimurium*					*Salmonella typhimurium*				
			TA 98	TA 100	TA 1535	TA 1537	TA 102	TA 98	TA 100	TA 1535	TA 1537	TA 102
Vehicle control	Vehicle control (dimethyl sulphoxide)	Mean	30.3	110.7	19.0	9.0	270.3	25.7	102.7	16.7	7.0	259.7
		±SD	1.5	2.5	1.0	1.0	2.3	1.2	5.5	0.6	1.0	5.0
	Lawn intensity		4+	4+	4+	4+	4+	4+	4+	4+	4+	4+
OLNP-18	4	Mean	29.3	108.3	19.0	9.3	265.3	26.7	99.3	16.7	7.0	263.3
		±SD	2.1	3.5	1.0	1.5	4.5	1.5	3.5	1.2	1.0	6.1
	Fold increase		1.0	1.0	1.0	1.0	1.0	1.0	1.0	1.0	1.0	1.0
	Lawn intensity		4+	4+	4+	4+	4+	4+	4+	4+	4+	4+
	13	Mean	29.7	112.7	18.3	9.7	266.7	27.0	101.3	16.3	7.3	260.0
		±SD	0.6	2.5	1.5	1.5	6.8	1.0	4.5	1.2	0.6	3.0
	Fold increase		1.0	1.0	1.0	1.1	1.0	1.1	1.0	1.0	1.0	1.0
	Lawn intensity		4+	4+	4+	4+	4+	4+	4+	4+	4+	4+
	40	Mean	30.0	109.3	18.3	9.3	270.7	25.0	101.3	16.0	7.0	260.7
		±SD	1.0	2.5	1.2	2.1	3.1	1.7	1.5	1.0	1.0	4.5
	Fold increase		1.0	1.0	1.0	1.0	1.0	1.0	1.0	1.0	1.0	1.0
	Lawn intensity		4+	4+	4+	4+	4+	4+	4+	4+	4+	4+
	130	Mean	30.7	108.0	18.7	8.7	266.3	25.7	98.7	16.0	7.3	262.0
		±SD	1.5	3.0	1.2	0.6	4.9	1.5	3.1	1.0	0.6	6.2
	Fold increase		1.0	1.0	1.0	1.0	1.0	1.0	1.0	1.0	1.0	1.0
	Lawn intensity		4+	4+	4+	4+	4+	4+	4+	4+	4+	4+
	400	Mean	19.3	79.0	14.0	4.7	248.3	18.3	75.0	9.7	3.3	240.3
		±SD	0.6	2.0	1.0	0.6	3.1	0.6	2.0	0.6	0.6	1.5
	Fold increase		0.6	0.7	0.7	0.5	0.9	0.7	0.7	0.6	0.5	0.9
	Lawn intensity		3+	3+	3+	3+	3+	3+	3+	3+	3+	3+
Positive control	100 *μ*L of respective positive control	Mean	391.0	396.7	147.3	121.0	615.0	372.7	388.0	141.7	112.0	602.7
		±SD	7.9	7.5	3.1	3.6	7.5	5.7	6.2	3.5	4.4	7.4
	Fold increase		12.9	3.6	7.8	13.4	2.3	14.5	3.8	8.5	16.0	2.3
	Lawn intensity		4+	4+	4+	4+	4+	4+	4+	4+	4+	4+

3+: Slightly thin lawn: distinguished by a noticeable thinning (slightly reduced) of the background lawn compared to vehicle control plates. 4+: thick lawn: distinguished by a healthy (normal) background lawn comparable to vehicle control plates. Values of revertants are in mean ± SD. Positive controls: for with S9: for *Salmonella typhimurium*, TA98, TA100, TA102, TA1535, and TA1537: 4 *μ*g/plate of 2-aminoanthracene. For S9: for TA98: 2 *μ*g/plate of 2-nitrofluorene. For TA100 and TA1535: 1 *μ*g/plate of sodium azide. For TA102: 0.5 *μ*g/plate of mitomycin C. For TA1537: 50 *μ*g/plate of 9-aminoacridine.

**Table 4:** Micronucleus data summary.

Sex	Group & dose (mg/kg)	PCE: total erythrocytes ratio	Total no. of PCE's scored	No. of micronucleus	% of micronucleus
Male	G1 & 0	0.52 ± 0.01	4017.20 ± 9.58	2.20 ± 0.84	0.05 ± 0.02
	G2 & 100 (CPA)	0.47^∗^ ± 0.01	4025.80 ± 1.30	30.00 ± 1.58	0.75^∗^ ± 0.04
	G3 & 2000	0.51 ± 0.01	4023.20 ± 7.98	2.40 ± 0.55	0.06 ± 0.01
Female	G1 & 0	0.51 ± 0.01	4013.20 ± 8.26	2.20 ± 0.84	0.05 ± 0.02
	G2 & 100 (CPA)	0.47^∗^ ± 0.01	4019.00 ± 7.31	28.40 ± 1.52	0.71^∗^ ± 0.04
	G3 & 2000	0.51 ± 0.01	4016.60 ± 6.88	2.60 ± 0.89	0.06 ± 0.02

CPA: cyclophosphamide monohydrate. ^∗^Statistically significant mean values with SD.

**(a):** 

Group, sex, & dose (mg/kg/day)		Total leucocyte count	Total erythrocyte count	Hemoglobin	Hematocrit	Mean corpuscular volume	Mean corpuscular hemoglobin	Mean corpuscular hemoglobin concentration
		(WBC)	(RBC)	(HGB)	(HCT)	(MCV)	(MCH)	(MCHC)
		(10^3^ cells/*μ*L)	(10^6^ cells/*μ*L)	(g/dL)	(%)	(fL)	(pg)	(g/dL)
G1, M, & 0	Mean	10.27	7.73	13.57	42.57	55.20	17.60	31.88
	±SD	2.48	0.74	1.13	3.35	1.85	0.75	0.61
	*n*	10	10	10	10	10	10	10
G2, M, & 2000	Mean	10.81	7.93	13.79	43.76	55.23	17.40	31.53
	±SD	2.05	0.68	1.08	3.45	1.43	0.59	0.80
	*n*	10	10	10	10	10	10	10
G1, F, & 0	Mean	8.86	7.22	13.38	40.97	56.81	18.59	32.72
	±SD	3.24	0.70	1.07	3.30	1.47	0.56	0.94
	*n*	10	10	10	10	10	10	10
G2, F, & 2000	Mean	8.38	7.15	13.05	40.85	57.13	18.26	31.98
	±SD	1.83	0.31	0.63	2.12	1.14	0.58	0.89
	*n*	10	10	10	10	10	10	10
G1R, M, & 0	Mean	8.77	8.10	14.38	42.80	52.82	17.76	33.56
	±SD	2.27	0.40	0.83	1.99	0.83	0.25	0.53
	*n*	5	5	5	5	5	5	5
G2R, M, & 2000	Mea*n*	11.29	8.28	14.68	43.58	52.76	17.76	33.66
	±SD	4.43	0.49	0.50	1.30	1.73	0.74	0.50
	*n*	5	5	5	5	5	5	5
G1R, F, & 0	Mean	7.84	7.63	13.90	41.14	53.98	18.24	33.80
	±SD	3.27	0.56	0.71	2.37	2.13	0.78	0.42
	*n*	5	5	5	5	5	5	5
G2R, F & 2000	Mean	6.63	7.54	13.42	41.40	55.02	17.88	32.46
	±SD	1.55	0.66	1.11	2.92	2.17	1.78	2.31
	*n*	5	5	5	5	5	5	5

M: male; F: female; R: recovery; SD: standard deviation; *n*: number of animals.

**(b):** 

		Platelet count	Mean platelet volume	Reticulocyte count	Neutrophils	Lymphocytes	Monocytes	Eosinophils	Basophils
Group, sex, & dose (mg/kg/day)									
		(PLT)	(MPV)	(Retic)	(Neut)	(Lymph)	(mono)	(Eos)	(Baso)
		(10^3^ cells/*μ*L)	(fL)	(%)	(%)	(%)	(%)	(%)	(%)
G1, M, & 0	Mean	860.10	6.71	2.07	23.52	70.72	3.31	1.14	0.27
	±SD	141.84	0.21	0.57	6.03	6.64	1.56	0.37	0.08
	*n*	10	10	10	10	10	10	10	10
G2, M, & 2000	Mean	957.90	6.85	1.68	22.46	72.51	2.25	0.96	0.35
	±SD	139.84	0.48	0.41	4.95	5.27	0.69	0.46	0.14
	*n*	10	10	10	10	10	10	10	10
G1, F, & 0	Mean	975.00	6.97	1.89	27.82	65.86	3.26	1.39	0.34
	±SD	160.33	0.65	0.37	8.24	8.16	0.56	0.49	0.08
	*n*	10	10	10	10	10	10	10	10
G2, F, & 2000	Mean	898.60	6.72	2.07	19.65^∗^	75.24^∗^	2.84	0.93	0.25^∗^
	±SD	103.71	0.30	0.76	3.38	3.94	0.91	0.51	0.08
	*n*	10	10	10	10	10	10	10	10
G1R, M, & 0	Mean	994.60	10.56	1.56	25.70	67.80	3.08	2.06	0.40
	±SD	128.40	0.23	0.42	6.01	6.07	0.53	0.71	0.10
	*n*	5	5	5	5	5	5	5	5
G2R, M, & 2000	Mean	977.20	10.42	1.27	30.82	61.02	5.84	1.24	0.42
	±SD	149.61	0.38	0.20	24.14	28.58	5.64	0.78	0.11
	*n*	5	5	5	5	5	5	5	5
G1R, F, & 0	Mean	1039.40	10.28	1.97	21.48	73.34	2.68	1.40	0.40
	±SD	109.56	0.57	0.86	6.31	7.56	1.12	0.48	0.14
	*n*	5	5	5	5	5	5	5	5
G2R, F, & 2000	Mean	992.20	10.52	1.35	27.14	66.42	3.80	1.62	0.36
	±SD	167.89	0.22	0.11	6.18	6.16	0.94	0.56	0.13
	*n*	5	5	5	5	5	5	5	5

M: male; F: female; R: recovery; SD: standard deviation; *n*: number of animals.

**(c):** 

Group, sex, & dose (mg/kg/day)		Absolute reticulocyte count	Absolute neutrophils	Absolute lymphocytes	Absolute monocytes	Absolute eosinophils	Absolute basophils	Prothrombin time	Activated prothrombin time
		(Retic)	(Neut)	(Lymph)	(Mono)	(Eos)	(Baso)	(PT)	(APTT)
		(10^9^ cells/L)	(10^3^ cells/*μ*L)	(10^3^ cells/*μ*L)	(10^3^ cells/*μ*L)	(10^3^ cells/*μ*L)	(10^3^ cells/*μ*L)	(seconds)	(seconds)
G1, M, & 0	Mean	158.20	2.46	7.24	0.32	0.12	0.03	28.69	30.46
	±SD	39.52	1.14	1.68	0.12	0.05	0.01	5.31	5.34
	*n*	10	10	10	10	10	10	10	10
G2, M, & 2000	Mean	133.39	2.41	7.87	0.24	0.10	0.04	23.30^∗^	30.82
	±SD	33.29	0.61	1.73	0.07	0.04	0.02	1.81	4.13
	*n*	10	10	10	10	10	10	10	10
G1, F, & 0	Mean	136.51	2.36	5.92	0.29	0.13	0.03	26.93	28.47
	±SD	28.71	0.73	2.62	0.14	0.07	0.02	4.27	3.67
	*n*	10	10	10	10	10	10	10	10
G2, F, & 2000	Mean	147.97	1.63^∗^	6.32	0.24	0.08	0.02	27.74	25.25
	±SD	53.11	0.37	1.54	0.10	0.05	0.01	4.00	3.62
	*n*	10	10	10	10	10	10	10	10
G1R, M, & 0	Mean	125.84	2.24	5.97	0.27	0.17	0.03	24.30	31.58
	±SD	32.73	0.62	1.77	0.07	0.05	0.01	2.47	2.36
	*n*	5	5	5	5	5	5	5	5
G2R, M, & 2000	Mean	105.54	3.47	6.84	0.71	0.13	0.05	22.84	29.66
	±SD	17.10	3.37	4.76	0.78	0.10	0.03	2.58	4.66
	*n*	5	5	5	5	5	5	5	5
G1R, F, & 0	Mean	151.86	1.61	5.85	0.20	0.11	0.03	21.90	32.54
	±SD	73.03	0.70	2.78	0.11	0.03	0.02	5.46	7.01
	*n*	5	5	5	5	5	5	5	5
G2R, F, & 2000	Mean	102.36	1.83	4.38	0.24	0.11	0.03	23.16	29.06
	±SD	15.44	0.69	1.06	0.03	0.05	0.01	1.75	4.46
	*n*	5	5	5	5	5	5	5	5

M: male; F: female; R: recovery; SD: standard deviation; *n*: number of animals.

**(a):** 

Group, sex, & dose (mg/kg/day)		Glucose	Urea	Creatinine	Total cholesterol	Triglycerides	High-density lipoprotein	Low-density lipoprotein	Total protein	Albumin	Alanine aminotrans ferase	Aspartate aminotrans ferase
		(GLU)		(CRE)	(CHO)	(TRI)	(HDL)	(LDL)	(TPR)	(ALB)	(ALT)	(AST)
		(mg/dL)	(mg/dL)	(mg/dL)	(mg/dL)	(mg/dL)	(mg/dL)	(mg/dL)	(g/dL)	(g/dL)	(U/L)	(U/L)
G1, M, & 0	Mean	98.80	32.66	0.54	41.60	34.10	8.76	5.20	6.41	2.87	59.30	101.10
	±SD	11.87	4.21	0.04	4.14	12.60	1.29	1.11	0.24	0.17	15.68	22.61
	*n*	10	10	10	10	10	10	10	10	10	10	10
G2, M, & 2000	Mean	102.20	37.23^∗^	0.54	40.20	24.30^∗^	8.72	5.59	6.25	2.93	49.10	94.50
	±SD	15.80	4.69	0.06	5.59	7.50	1.41	1.20	0.33	0.17	8.72	16.51
	*n*	10	10	10	10	10	10	10	10	10	10	10
G1, F, & 0	Mean	109.70	36.05	0.61	47.80	25.70	12.91	2.23	6.76	3.23	40.30	93.00
	±SD	11.04	7.28	0.04	10.17	10.48	2.72	0.57	0.46	0.28	7.17	10.79
	*n*	10	10	10	10	10	10	10	10	10	10	10
G2, F, & 2000	Mean	107.70	36.29	0.61	53.20	23.90	14.29	3.28^∗^	7.18^∗^	3.50^∗^	36.80	87.50
	±SD	11.37	5.11	0.02	6.36	12.85	1.85	0.70	0.31	0.29	11.65	20.48
	*n*	10	10	10	10	10	10	10	10	10	10	10
G1R, M, & 0	Mean	77.80	31.38	0.55	40.40	33.60	9.16	4.90	6.72	2.98	73.80	111.60
	±SD	5.02	4.88	0.01	4.77	8.32	1.11	0.99	0.29	0.09	11.67	20.23
	*n*	5	5	5	5	5	5	5	5	5	5	5
G2R, M, & 2000	Mean	84.80	31.58	0.57	42.20	30.40	9.08	5.26	6.80	3.10	61.20	110.80
	±SD	6.83	3.34	0.04	12.30	4.10	2.53	1.82	0.19	0.13	10.62	17.37
	*n*	5	5	5	5	5	5	5	5	5	5	5
G1R, F, & 0	Mean	108.80	31.52	0.64	44.80	39.20	12.58	2.60	7.14	3.68	47.40	96.60
	±SD	13.61	7.39	0.06	5.07	18.63	1.49	0.38	0.54	0.29	6.80	9.89
	*n*	5	5	5	5	5	5	5	5	5	5	5
G2R, F, & 2000	Mean	117.20	36.28	0.60	51.00	28.60	13.44	3.24^∗^	6.88	3.51	41.20	105.40
	±SD	16.13	3.88	0.09	14.87	6.54	3.58	0.44	0.28	0.19	12.09	25.17
	*n*	5	5	5	5	5	5	5	5	5	5	5

**(b):** 

Group, sex, & dose (mg/kg/day)		Alkaline phosphatase	Total bilirubin	Calcium	Phosphorous	Cholinesterase	Globulin	Albumin/globulin ratio	Blood urea nitrogen	Sodium	Potassium	Chloride
		(ALP)	(BIT)	(CAL)	(PHO)	(CHE)	(GLO)	(A/G ratio)	(BUN)	(Na)	(K)	(CLO)
		(U/L)	(mg/dL)	(mg/dL)	(mg/dL)	(U/L)	(g/dL)		(mg/dL)	(mmol/L)	(mmol/L)	mmol/L)
G1, M, & 0	Mean	129.90	0.12	7.95	8.73	167.40	3.54	0.82	15.24	144.96	4.00	116.49
	±SD	31.96	0.02	0.51	2.29	29.67	0.25	0.09	1.96	1.90	0.36	2.28
	*n*	10	10	10	10	10	10	10	10	10	10	10
G2, M, & 2000	Mean	108.10	0.12	7.80	7.42	173.60	3.32	0.89	17.38∗	146.52∗	4.21	116.04
	±SD	35.41	0.03	0.62	1.61	39.64	0.26	0.08	2.19	1.36	0.27	2.46
	*n*	10	10	10	10	10	10	10	10	10	10	10
G1, F, & 0	Mean	58.10	0.14	8.32	8.83	708.90	3.53	0.91	16.82	145.94	3.45	117.93
	±SD	11.45	0.02	0.51	3.07	169.22	0.20	0.05	3.39	1.65	0.27	3.18
	*n*	10	10	10	10	10	10	10	10	10	10	10
G2, F, & 2000	Mean	55.80	0.14	8.89^∗^	6.78	704.20	3.68	0.96	16.94	145.23	3.82^∗^	115.96
	±SD	15.02	0.03	0.53	2.82	120.23	0.19	0.10	2.39	1.10	0.34	1.70
	*n*	10	10	10	10	10	10	10	10	10	10	10
G1R, M, & 0	Mean	128.40	0.00	9.20	4.92	89.20	3.74	0.80	14.64	141.30	3.24	105.20
	±SD	30.47	0.00	0.38	0.29	26.03	0.22	0.04	2.28	1.44	0.20	2.49
	n	5	5	5	5	5	5	5	5	5	5	5
G2R, M, & 2000	Mean	120.00	0.00	9.26	4.94	108.60	3.70	0.84	14.74	141.60	3.44	105.90
	±SD	24.81	0.00	0.26	0.50	28.81	0.29	0.10	1.56	1.53	0.15	1.32
	*n*	5	5	5	5	5	5	5	5	5	5	5
G1R, F, & 0	Mean	44.00	0.00	9.40	3.52	871.80	3.46	1.07	14.71	141.52	3.14	107.44
	±SD	10.93	0.00	0.34	0.26	196.78	0.33	0.10	3.45	0.54	0.25	0.53
	*n*	5	5	5	5	5	5	5	5	5	5	5
G2R, F, & 2000	Mean	54.80	0.00	9.46	3.90	821.00	3.37	1.05	16.93	141.62	3.03	106.86
	±SD	21.73	0.00	0.09	0.42	386.50	0.29	0.13	1.81	0.49	0.33	1.15
	*n*	5	5	5	5	5	5	5	5	5	5	5

**Table 7:** Absolute organ weights (male).

Group, sex ,& dose (mg/kg/day)		Adrenals	Thymus	Spleen	Testes	Epididymis	Heart	Kidneys	Brain	Liver	Prostate+seminal vesicles with coagulating glands (PSC)	Lungs	Thyroid along with parathyroid	Pituitary gland
G1, M, & 0	Mean	0.0608	0.3400	0.7810	3.3092	1.4409	1.2944	2.6879	2.1109	11.5935	3.2575	2.6675	0.0242	0.0164
	±SD	0.0091	0.0463	0.1151	0.1730	0.1076	0.0921	0.2012	0.1088	0.8979	0.1458	0.2396	0.0059	0.0022
	*n*	10	10	10	10	10	10	10	10	10	10	10	10	10
G2, M, & 2000	Mean	0.0565	0.3344	0.7248	3.1445	1.2445^∗^	1.3274	2.7383	2.0560	11.7990	3.2737	2.6360	0.0225	0.0158
	±SD	0.0086	0.0336	0.0975	0.2906	0.1881	0.1216	0.2304	0.1050	0.9035	0.2577	0.2285	0.0035	0.0018
	*n*	10	10	10	10	10	10	10	10	10	10	10	10	10
G1R, M, & 0	Mean	0.0512	0.3591	0.5886	3.5400	1.4742	1.3123	2.8897	2.0364	11.2755	2.9858	2.8537	0.0269	0.0166
	±SD	0.0026	0.0622	0.0784	0.1882	0.0952	0.0437	0.2621	0.0866	1.3584	0.1103	0.0579	0.0035	0.0015
	*n*	5	5	5	5	5	5	5	5	5	5	5	5	5
G2R, M, & 2000	Mean	0.0521	0.3399	0.6278	3.4821	1.4857	1.3230	2.9077	1.9948	10.9147	3.1913	2.8180	0.0253	0.0173
	±SD	0.0065	0.0375	0.0601	0.1046	0.0416	0.0573	0.1115	0.0294	1.2758	0.2826	0.1193	0.0031	0.0012
	*n*	5	5	5	5	5	5	5	5	5	5	5	5	5

**Table 8:** Absolute organ weights (female).

Group, sex, & dose (mg/kg/day)		Adrenals	Thymus	Spleen	Ovaries	Uterus	Heart	Kidneys	Brain	Liver	Lungs	Thyroid along with parathyroid	Pituitary gland
G1, F, & 0	Mean	0.0657	0.3509	0.7394	0.1568	0.5757	1.0963	1.8356	1.9791	7.9013	2.1024	0.0268	0.0188
	±SD	0.0065	0.0421	0.2297	0.0105	0.0865	0.0752	0.1306	0.0434	0.4640	0.1862	0.0050	0.0027
	*n*	10	10	10	10	10	10	10	10	10	10	10	10
G2, F, & 2000	Mean	0.0695	0.3513	0.7323	0.1449	0.6136	1.0601	1.8155	1.9623	8.8766^∗^	2.1036	0.0251	0.0175
	±SD	0.0051	0.0347	0.1299	0.0173	0.0774	0.0873	0.1235	0.0449	0.9546	0.1393	0.0050	0.0027
	*n*	10	10	10	10	10	10	10	10	10	10	10	10
G1R, F, & 0	Mean	0.0673	0.2904	0.6808	0.1301	0.8605	0.9707	1.7914	1.9489	7.4328	1.9085	0.0272	0.0182
	±SD	0.0042	0.0179	0.0641	0.0080	0.1208	0.0734	0.1118	0.1062	0.4430	0.1741	0.0023	0.0027
	*n*	5	5	5	5	5	5	5	5	5	5	5	5
G2R, F, & 2000	Mean	0.0764	0.2769	0.6925	0.1429	0.7593	0.9523	1.8887	1.9805	7.7799	2.0088	0.0267	0.0186
	±SD	0.0101	0.0231	0.1331	0.0280	0.0497	0.0601	0.2049	0.0988	0.9160	0.1576	0.0018	0.0013
	*n*	5	5	5	5	5	5	5	5	5	5	5	5

## Data Availability

Data supporting the results of this study can be shared by contacting the first author, Somashekara Nirvanashetty Ph.D at soma@olenelife.com.
